# Seroprevalence of HCV and HIV Infections by Year of Birth in Spain: Impact of US CDC and USPSTF Recommendations for HCV and HIV Testing

**DOI:** 10.1371/journal.pone.0113062

**Published:** 2014-12-01

**Authors:** Alvaro Mena, Luz Moldes, Héctor Meijide, Angelina Cañizares, Ángeles Castro-Iglesias, Manuel Delgado, Sonia Pértega, José Pedreira, Germán Bou, Eva Poveda

**Affiliations:** 1 Grupo de Virología Clínica, Instituto de Investigación Biomédica de A Coruña-Complexo Hospitalario Universitario de A Coruña, Universidade da Coruña, A Coruña, Spain; 2 Servicio de Microbiología, Instituto de Investigación Biomédica de A Coruña-Complexo Hospitalario Universitario de A Coruña, Universidade da Coruña, A Coruña, Spain; 3 Service of Internal Medicine, Hospital Quiron, A Coruña, Spain; 4 Servicio de Aparato Digestivo, Instituto de Investigación Biomédica de A Coruña-Complexo Hospitalario Universitario de A Coruña, Universidade da Coruña, A Coruña, Spain; 5 Unidad de Epidemiología Clínica y Bioestadística, Instituto de Investigación Biomédica de A Coruña-Complexo Hospitalario Universitario de A Coruña. Universidade da Coruña, A Coruña, Spain; Saint Louis University, United States of America

## Abstract

**Background:**

The US Centers for Disease Control and Prevention (CDC) recently add the advice of one-time testing of HCV infection in persons born during 1945–1965. Moreover, the US Preventive Services Task Force (USPSTF) newly recommended one-time HIV testing for persons aged 15–65. Herein, we evaluate the potential impact of these recommendations in a reference medical area of Spain.

**Methods:**

All assays results entries for HCV and HIV serological markers ordered at a reference lab from primary care and specialized physicians between 2008 and 2012 were recorded in a medical area which covers 501,526 citizens in Northern Spain. The year of birth were also documented.

**Results:**

A total of 108,159 anti-HCV-Ab results were generated during the study period. The global rate of anti-HCV-Ab+ was 7.7% (95% CI: 7.6%–7.9%), being more prevalent in men than women (8.6% vs. 4.5%). By year of birth, the highest prevalence was found in persons born between 1955 and 1970. HCV genotype 1 was the most prevalent (59.7%) followed by genotype 3 (22.7%). Regard HIV infection, among 65,279 anti-HIV results generated the prevalence of anti-HIV+ was 1.1% (95% CI: 1.0%–1.2%), being more frequent in men (2% vs 0.5%). The years of birth with highest rates of HIV infection exactly match with those for HCV infection.

**Conclusions:**

The highest rates of HCV and HIV infections are found between 1960 and 1965. Different historical and social circumstances such as the huge intravenous drug use epidemic in the eighties in Spain, might explain it. Therefore, each country needs to determine its own HCV and HIV seroprevalences by year of birth to establish the proper recommendations for the screening of both infections.

## Introduction

Hepatitis C Virus infection (HCV) is considered a global public health problem causing high rates of morbidity and mortality worldwide. The World Health Organization (WHO) has estimated that around 2–3% of the world's population are infected with HCV which imply that 120–170 million people are HCV chronically infected [1], [2].

Approximately 75–85% of acute HCV-infections progress to chronic liver disease [3]. Since acute and chronic HCV infections are frequently asymptomatic, patients frequently are unaware they are infected until very advanced stages of their disease [4]. After 30 years of HCV infection, around 15%–30% of patients develop cirrhosis and 1%–3% hepatocellular carcinoma (HCC), which is the fastest growing cause of cancer-related mortality. Of note, HCV infection accounts for approximately 50% of incident HCC [5]. Due to this latency, despite HCV incidence has decreased in last decades, HCV-related mortality is increasing annually. According to the Global Burden of Disease estimates, hepatitis B and hepatitis C caused together 1.4 million deaths per year, including deaths from acute infection, liver cancer and cirrhosis, very close to HIV infection (1.7 million deaths/year) or tuberculosis (1.4 million deaths/year) in the worldwide [6]. In the United States, HCV-associated mortality recently has exceeded the number of deaths attributed to HIV [7]. A recent study carried in the United States estimated that the mortality rate in HCV-infected persons is up to 12 times higher than that found in the general population [8].

In 2012, the recommendation of Centers for Disease Control and Prevention (CDC) expanded their recommendations for HCV testing with a birth-year-based strategy. In 1998, CDC recommended routine HCV testing for high-risk populations: exposure to injected illegal drugs, patients in hemodialysis or recipients of organ transplants or transfusion previous to 1992, HIV+ patients, clinical suspicion and persons with recognized exposure (Health Care workers and children born to HCV+ women) [9]. National Health and Nutrition Examination Survey (NHANES) data analysis estimated that the prevalence of HCV infection was 5-fold greater in persons born during 1945 to 1965 (3.25%) than other adults; this age group represents 76.5% of the total HCV-prevalence and 73% of HCV-related mortality in the United States [10], [11], thus CDC is now recommending one-time HCV testing for persons born during these years [12].

HIV infection continues being an important public health problem. In Spain, access to the health service is universal and free. It has been estimated that 72% of people receive health care at least once a year [13]; but the late presenters are about 40% of newly diagnoses patients [13], similar to other high-income countries [14]. Two third of this late presenters had one or more contacts with the health service previously, recognized as a missed opportunity for earlier HIV diagnosis [15]. Screening strategies are critical to reduce the number of undiagnosed persons and the proportion of late diagnoses in order to modify the course of the epidemic. Recently, the US Preventive Services Task Force (USPSTF) advised once HIV testing for persons aged 15–65 [16]; in 2010. Moreover, the European Centre for Disease Control (ECDC), recommended the promotion of strategies aimed at key populations at risk to enable early diagnosis of infection [17].

In this context, the aim of this study was to assess the seroprevalence of HCV and HIV infections by year of birth in our population to analyze the potential impact of these recommendations in Northern Spain.

## Methods

### Study population

This cross-sectional study was carried out in Northern Spain (A Coruña University Hospital), which covers a medical care area of 501.526 citizens. All assays results entries for HCV and HIV serological markers ordered at our laboratory from primary care and specialized physicians between 2008 and 2012 were recorded. Demographic data (sex and year of birth) and laboratory parameters (anti-HCV, HCV genotype, HCV-RNA, anti-HIV, HIV-RNA and CD4 count), considering the first determination after the positive serology result, were also documented. All HIV/HCV coinfected patients followed in the same area were also recorded for the analysis.

The study protocol was reviewed and approved by the Medical Ethics Committee of the University Hospital of A Coruña. Publication of the study data is in accordance with the community standards and approved by the ethics committee. All patients data were anonymized and de-identified prior to analysis. The identification numbers of patients were recoded blindly.

### Serological tests

All serological tests were done with VITROS 3600 Immunodiagnostic System (Ortho Clinical Diagnostics, Inc., a Johnson & Johnson company, NY, USA) using: Anti-HIV 1+2 Reagent Pack, and Anti-HCV Reagent Pack, all of them VITROS Immunodiagnostic Products. All patients with anti-HCV or anti-HIV antibodies positive sera were retested using INNO-LIA HCV Score and INNO-LIA HIV I/II Score respectively by Auto-LIA 48 (Innogenetics N.V. Ghent, Belgium) as complementary tests. Patients with both anti-HCV and LIA positive reports were considered HCV positive. And patients with both anti-HIV and LIA positive reports were considered HIV positive.

Plasma HCV-RNA was quantified using the real-time polymerase chain reaction (RT-PCR) assay Cobas AmpliPrep/CobasTaqMan HCV QuantitativeTest, version 2.0. (Roche Diagnostics. Ltd., in Rotkreuz, Switzerland) with a lower limit of detection of 10 IU/mL. Plasma HIV-RNA was quantified using the RT-PCR assay Cobas AmpliPrep/CobasTaqMan HIV-1 Test, version 2.0. (Roche Diagnostics. Ltd., in Rotkreuz, Switzerland) with a lower limit of detection of 20 copies/mL. HCV genotyping was measured with a commercial linear array hepatitis C virus genotyping test and linear array detection kit (Roche Molecular Systems, Inc. Branchburg, USA). All assays were performed according to the manufacturer's instructions.

Of note, to avoid an overestimation of both infections all duplicated HCV and HIV tests for a same patient were identified and excluded for the analysis.

### Statistical analyses

Anti-HCV+ and anti-HIV+ seroprevalences were calculated, according to year of birth, together with their 95% confidence intervals (CI). Quantitative data were expressed as mean (standard deviation) or median (interquartile range) and analyzed with t-Student test. Qualitative data were expressed as percentage and compared with chi-square test.

A time-trend joinpoint regression analysis was performed to analyze trends in seroprevalence data according with year of birth. This technique provides the estimated annual percent change (APC) and allows detecting points in time at which significant changes in the trends occur. For each APC estimate, 95% confidence interval was also calculated.

Results were considered statistically significant when p<0.05. Statistical programs utilized were SPSS v16.0 for Windows (SPSS Inc.,Chicago,IL) and Joinpoint Regression Program, version 3.4.2.

## Results

### 1.- HCV infection

#### a) Seroprevalence of HCV infection

A total of 108,159 anti-HCV results were generated during the study period, the mean age was 45.3±18.0 years and 51.8% were male. The overall anti-HCV+ rate were 7.7% (95% CI: 7.6%–7.9%). The yearly prevalence has remained stable during the last 5 years: 9.1% (2008), 8.0% (2009), 7.3% (2010), 6.8% (2011), and 7.9% (2012).

**Figure 1:**
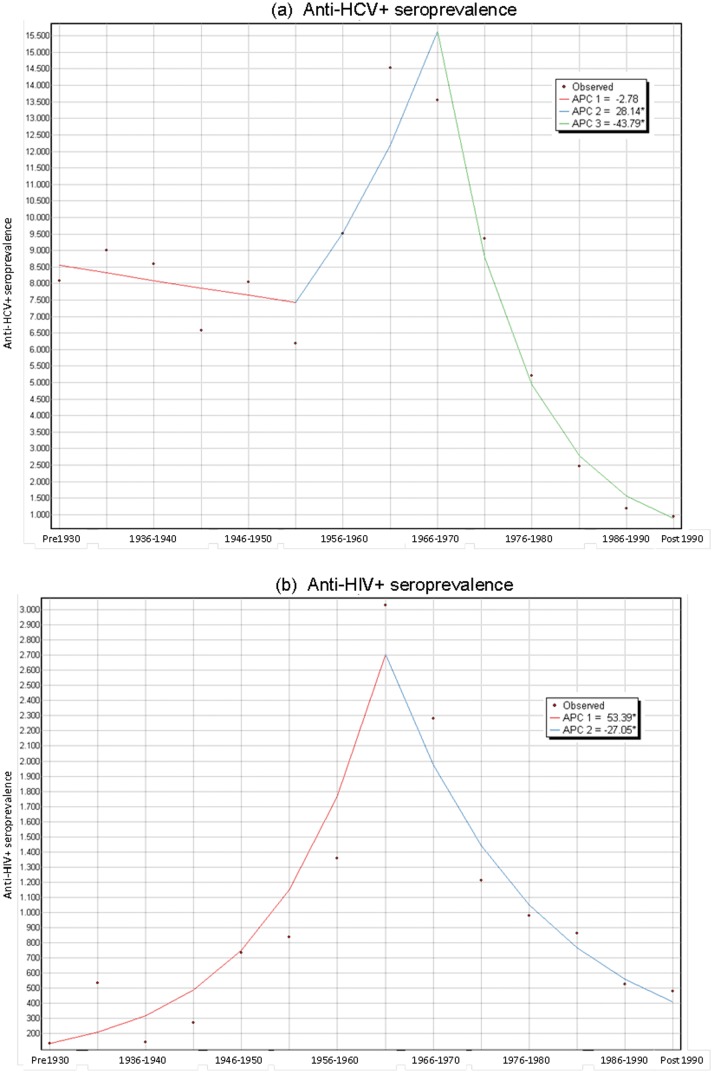
Joinpoint regression analysis: trends in HCV and HIV seroprevalence according to year of birth.

Table 1 depicts the rates of anti-HCV+ by year of birth. In the joinpoint regression analysis, three different trends were observed. Anti-HCV+ seroprevalence remained stable among those born before 1955, followed by an increase among those born between 1955 and 1970 (APC = 28.14%). A significant decrease was documented for those born afterwards (APC = −43.8%) (Table 2, Figure 1).

**Table 1:** Seroprevalence of HCV and HIV infections, by year of birth, from 2008 to 2012.

Year of birth	Tested for anti-HCV Ab	Anti-HCV+		Tested for anti-HIV Ab	Anti-HIV+	
	n	n (%)	95% CI	n	n (%)	95% CI
Pre 1930	4131	334 (8.1)	7.2–8.9	1487	2 (0.1)	0.0–0.5
1931–1935	4306	388 (9)	8.1–9.9	1496	8 (0.5)	0.1–0.9
1936–1940	4294	369 (8.6)	7.7–9.4	1406	2 (0.1)	0–0.5
1941–1945	5498	362 (6.6)	5.9–7.2	1844	5 (0.3)	0.1–0.6
1946–1950	7839	630 (8)	7.4–8.6	2182	16 (0.7)	0.3–1.1
1951–1955	6652	411 (6.2)	5.6–6.8	2387	20 (0.8)	0.4–1.2
1956–1960	8361	795 (9.5)	8.8–10.1	3311	45 (1.4)	0.9–1.8
1961–1965	8881	1290 (14.5)	13.8–15.3	4027	122 (3)	2.5–3.6
1966–1970	11167	1513 (13.5)	12.9–14.2	6582	150 (2.3)	1.9–2.6
1971–1975	13113	1227 (9.4)	8.7–9.7	10654	129 (1.2)	1.0–1.4
1976–1980	13315	693 (5.2)	4.8–5.6	12665	124 (1)	0.8–1.1
1981–1985	9968	245 (2.5)	2.1–2.8	9031	78 (0.9)	0.7–1.1
1986–1990	5724	68 (1.2)	0.9–1.5	4759	25 (0.5)	0.3–0.7
Post 1990	4910	46 (0.9)	0.6–1.2	4170	20 (0.5)	0.2–0.7
TOTAL	108159	8371 (7.7)	7.6–7.9	65279	742 (1.1)	1.0–1.2

**Table 2:** Joinpoint regression analysis: trends in HCV and HIV seroprevalence according to year of birth.

	Period 1		Period 2		Period 3	
	Year of birth	APC	Year of birth	APC	Year of birth	APC
Anti-HCV+	Pre 1930–1955	−2.8 (−9.8;4.8)	1955–1970	28.1 (2.4;60.4)	1970-Post 1990	−43.8 (−49.8;−37.1)
Anti-HIV+	Pre 1930–1965	53.4 (26.5;85.9)	1965- Post 1990	−27.1 (−34.0;−19.3)	-	-

APC = *Annual percent change*.

#### b) Clinical and microbiological characteristics of anti-HCV+ patients

Mean age on anti-HCV+ patients was 50.2±16.6 years and 66.4% were male. The HCV prevalence in men was 9.9% (95% CI: 9.7–10.2) and 5.4% (95% CI: 5.2–5.6) (OR 1.9, 95%CI: 1.8–2.0; p<.001) in women; who were 10.8 years older than men (57.4±18.5 vs 46.6±14.2; p<.001, 95%CI: 10.0–11.6).

From anti-HCV+ individuals, HCV-RNA was performed in 6442 (77%), a mean of 44.2±25.4 months after positive serology; of them, 56.3% (95% CI: 55.1%–57.5%) had detectable RNA-HCV, with a mean value of 5.7±1.1 IU/mL. HCV genotype was documented in 929 individuals, with the following distribution: G1 59.7% (95% CI: 56.6%–62.9%), G2 5.4% (95% CI: 4.1%–7.0%), G3 22.7% (95% CI: 20.1%–25.5%), and G4 12.2% (95% CI: 10.2%–14.4%); more than two-third had genotypes 1 or 4.

### 2.- HIV infection

#### a) Seroprevalence of HIV infection

A total of 65,279 anti-HIV tests were performed between 2008 and 2012. The mean age of tested was 39.5±16.1 years and 44% were male. The prevalence of anti-HIV+ was 1.1% (95% CI: 1.0%–1.2%). The yearly rate has remained stable during the study period: 1.1% (2008), 1.3% (2009), 1.4% (2010), 0.8% (2011), 1.0% (2012).

Similarly to HCV seroprevalence, the highest rates of anti-HIV+ Ab was observed in the period between 1961–1965 (3%), followed by the period between 1966–1970 (2.3%) (Table 1). In this case, the joinpoint regression analysis identified two different trends. Anti-HIV+ seroprevalence increased significantly with year of birth for those born before 1965 (APC = 53.4%), decreasing afterwards with an APC = −27.1% (Table 2, Figure 1).

#### b) Clinical and microbiological characteristics of anti-HIV+ patients

The prevalence of anti-HIV+ in men was 2.0% (95% CI: 1.8–2.1) and in women.5% (95% CI:.4–.6) (OR 4.1, 95%CI: 3.5–4.8; p<.001), mean age 39.5±11.0, without difference between men (39.9±10.7 years) and women (38.5±11.8 years) (95% CI −3.5–.7; p = .19).

Overall 742 were anti-HIV+, 310 (43%) were newly diagnosed. The mean age of newly HIV diagnosed patients was 38.5±11.3 years and 82% were men. The median CD4 counts were 361 cels/µl (126–563) and the median HIV-RNA was 5 log cop/mL (4.4–5.5). The CD4 counts was analysed a mean of 3.9±2.1 months after positive HIV serology, and HIV-RNA a mean of 4.2±2.2 months after. Of them, 50.5% were late presenters (AIDS defining illness or <350 cells/µl total CD4 count). The prevalence of anti-HCV+ Ab in this subgroup of newly diagnosed was 13%.

**Figure 2:**
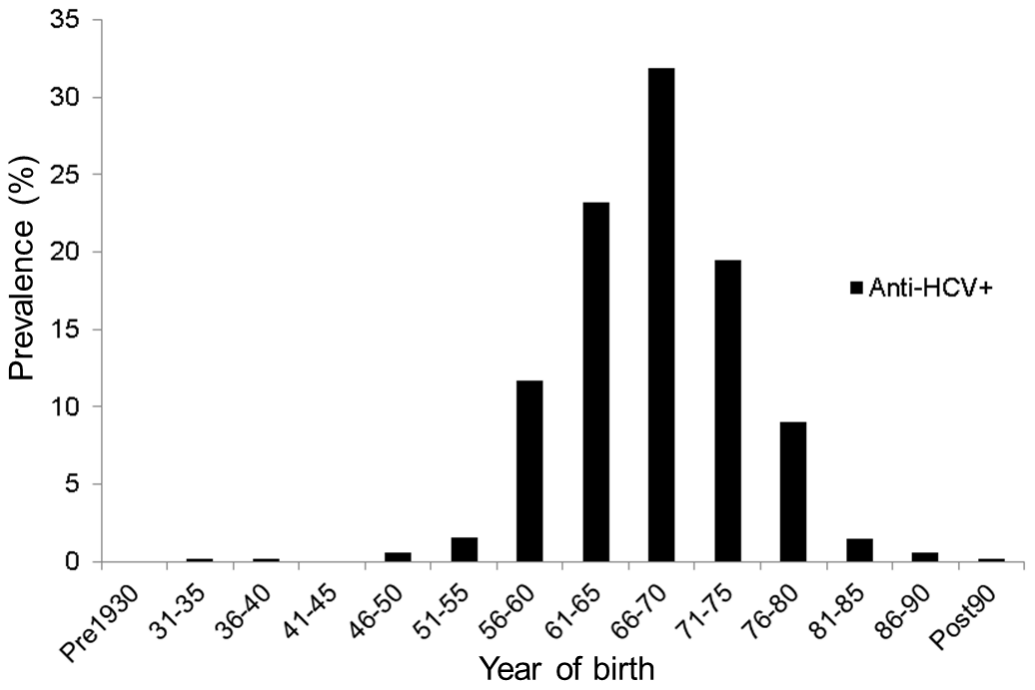
Distribution of the HCV/HIV co-infected patients (n = 553) by year of birth.

A total of 1300 HIV infected patients were on clinical follow-up within the study period in this medical area. The prevalence of HCV/HIV co-infection was 54%. Figure 2 illustrates the distribution by year of birth of this HCV/HIV co-infected population.

## Discussion

This study evaluates the seroprevalence of HCV and HIV infections by year of birth in a medical reference area of Northern Spain between 2008 and 2012. The overall seroprevalence of HCV infection during the study period was 7.7%. It is significantly higher than previously reported in different studies in Spain in general population and blood donors cohorts, with rates around 2.5–2.9% [18], [19]. The higher prevalence we observed might be explained because our study population includes patients with medical care requirements. Therefore, the HCV screening ordered to our laboratory is derived from patients attending to primary care clinics but also in clinical follow-up for hepatic disorders or previously diagnosed for HCV and/or HIV infections at A Coruña University Hospital. Moreover, HCV screening is also performed in patients attending at alcohol and drug detox centers. In all these populations is well-known the higher rates of HCV infection and consequently the seroprevalence of HCV infection is higher compared to the general population [20], [21]. The prevalence on anti-HCV+ was higher (OR 1.9) among men than in women and they were significantly younger. These findings might be explained by the higher use of intravenous drugs in men than in women.

The seroprevalence of HCV infection by year of birth highlights some differences with that reported from the US. While in our study population we found the highest seroprevalence of HCV infection in persons born during 1960–1970 (14%) in the NHANES cohort, in the same period, they observed a prevalence of 0.7%, more than three times lower. Therefore if a birth-year-based HCV-screening strategy was implemented in our country, these data must be considered to properly establish the optimal target population for HCV screening. Considering the CDC recommendation, HCV testing for persons born between 1945 and 1965, only 37% (n = 3126) of the total anti-HCV+ subjects (n = 8371) in our study population would be diagnosed [12]. Of the 63% hypothetical undiagnosed patients, 72% of them would have been born after 1965, mainly between 1965 and 1975, representing a younger group of patients in whom monitoring and treatment options are more effective. Historical and social circumstances might explain these differences including the huge intravenous drug use epidemic occurred in the eighties in Spain, probably some years after than in the US or other European countries. Regarding the virologic characteristics of HCV infection, nearly of 60% of patients had detectable HCV-RNA, half of them with high viral load (>6 log IU/mL) and more than two-third had genotypes 1 or 4. This data has important clinical and therapeutics implications since both genotypes are classically recognized such as the more difficult to treat. HCV genotypes distribution in our study is similar to that reported previously in the Spanish population [22]; in HCV/HIV coinfected we found a slightly higher prevalence for genotypes 2 and 3 than in HCV monoinfected, mainly associated with a higher proportion of intravenous drug users in this population. The HCV genotypes distribution in Spain is quite different than other European countries; of note, in Eastern Europe and UK the prevalence of genotype 3 is up to 50%, and in Italy the prevalence of genotype 2 is around 25% [18].

The new scenario for the treatment of HCV infection with new direct-acting antiviral agents (DAA) allowing significantly higher rates of cure for all genotypes and fairly or null adverse events, turns HCV diagnosis and treatment a cost-effective strategy [23]. However, in view of the results obtained in this study, each country must assess the HCV seroprevalence by year of birth to establish the optimal target population for HCV screening and to know its own HCV prevalence distribution. These preliminary data suggest as target population for HCV screening in Spain those persons born during 1955 and 1975.

The HIV seroprevalence was also evaluated since both infections share routes of transmission. The prevalence of anti-HIV+ Ab was 1.1%, with a comparatively high proportion of female's tested (56%), consistent with recommendations for prenatal HIV testing. However, the seroprevalence for HIV-infection is four times higher in men than in women, according with data provided by the Spanish Public Health authorities and the ECDC [24], [25]. Interestingly, the highest prevalence of HIV infection by year of birth exactly matches with the peak of HCV prevalence, recognized in persons born between 1955 and 1975. Moreover, these data support the hypothesis that the differences in the HCV prevalence distribution by year of birth in Spain compared with the US might be explained by the intravenous drug use epidemic occurred in the eighties in our country. Of note, in the last years, the main route of HIV transmission in our health area is sexually (more than 90% of newly HIV-infected) [26], with a mean age of 38 years old. Consequently, the prevalence of HIV infection is increasing in younger people. These data should be considered for the implementation of appropiate HIV screening programs.

Since this is a retrospective study including the results obtained from HCV and HIV screening in the last 5 years in our medical care area including high risk populations, the figure of HCV prevalence might be overestimated. However, the study has been conducted in a big sample (108,159 HCV tests, and 65,279 HIV tests). Moreover, concordant results regarding the anti-HCV+, anti-HIV+ and HCV/HIV co-infected prevalence distribution by year of birth support the accuracy of these results.

In summary, the prevalence of HCV and HIV distribution varies by year of birth in our population. The highest rates of HCV and HIV infection are observed in persons born between 1955–1975. The huge intravenous drug use epidemic occurred in the eighties in Spain might explain these findings. Of note, regional differences regard HIV and HCV prevalence and exposure groups have been report from European cohorts [27], [28] providing important information for future testing initiatives. Therefore, the recommendations made by the US CDC of HCV testing among persons born in 1945–1965 once in a lifetime, might not be applicable to other populations. For this reason, similarly to the US Health Institutions, each country needs to determine its own HCV and HIV seroprevalence by year of birth to identify its target population to implement the appropriate screening programs. In Spain, once in a lifetime HCV testing might be especially considered in persons aged 38–58. Regarding to HIV testing, due to the high number of new HIV infections observed in younger persons, and similarly to the USPSTF recommendations, once in a lifetime HIV testing might be considered in persons aged 15–65 in Spain.
